# Trimethoprim Antibiotic Adsorption from Aqueous Solution onto Eco-Friendly Zr-Metal Organic Framework Material

**DOI:** 10.3390/ma14247545

**Published:** 2021-12-08

**Authors:** Marwa Elkady, Kamal E. Diab, Hassan Shokry

**Affiliations:** 1 Chemical and Petrochemical Engineering Department, Egypt-Japan University of Science and Technology (E-JUST), New Borg El-Arab City, Alexandria 21934, Egypt; 2 Fabrication Technology Department, Advanced Technology and New Materials Research Institute (ATNMRI), City of Scientific Research and Technological Applications (SRTA-City), New Borg El-Arab City, Alexandria 21934, Egypt; 3 Nanoscience Department, Institute of Basic and Applied Sciences, Egypt-Japan University of Science and Technology, New Borg El-Arab City, Alexandria 21934, Egypt; kamal.essam@ejust.edu.eg; 4 Environmental Engineering Department, Egypt-Japan University of Science and Technology (E-JUST), New Borg El Arab City, Alexandria 21934, Egypt; 5 Electronic Materials Researches Department, Advanced Technology and New Materials Research Institute (ATNMRI), City of Scientific Research and Technological Applications (SRTA-City), New Borg El-Arab City, Alexandria 21934, Egypt

**Keywords:** zirconium Bio-MOF, trimethoprim, adsorption process, antibiotic decontamination, ecofriendly materials

## Abstract

The synthesis of Bio-MOF using aspartic acid as an organic linker and water as a solvent was performed to create an environmentally friendly material. The chemical composition, structure, and morphology of the synthesized zirconium Bio-MOF (MIP-202) was evaluated using X-ray diffraction (XRD), energy dispersive X-ray (EDX) spectroscopy, transmission electron microscopy (TEM), scanning electron microscopy (SEM), and X-ray photoelectron spectroscopy (XPS). The synthesized Bio-MOF was used as an adsorbent for trimethoprim antibiotic as pollutants from an aqueous solution under various operating parameters. The increase in the initial trimethoprim concentration from 2.5 mg/L to 20 mg/L decreased the decontamination efficiency from 77.6% to 35.9% at a solution pH of 7 with 0.5 g/L adsorbent dose after 60 min reaction time. The rise of adsorbent dose from 0.1 g/L to 1.5 g/L increased the removal efficiency from 47.7% to 87.6%. The maximum trimethoprim removal efficiency of 95% was attained at a solution pH of 11. Langmuir and pseudo-second order models described the adsorption process of trimethoprim antibiotic onto zirconium Bio-MOF and the chemo-physical nature of trimethoprim adsorption onto the synthesized zirconium Bio-MOF. Accordingly, it was evident that the prepared zirconium Bio-MOF (MIP-202) is an ecofriendly and efficient adsorbent for antibiotic decontamination from polluted water.

## 1. Introduction

Metal−organic frameworks (MOFs) are constructed by linking metal ions with organic linkers [1]. MOFs are characterized by the large surface area, controllable size/shape, uncommon physiochemical properties and high porosity [2]. According to the aforementioned characteristics, MOFs have been investigated for many applications such as gas storage [3], sensors [4], separation [5], catalysis [6], drug delivery [7] and water purification [8]. However, MOFs have some shortcomings such as insufficient stability and a small pore size, which hinder their application for water treatment [9]. Adsorption is considered one of the auspicious processes for water purification due to its simplicity [10]. Moreover, this process does not produce any secondary pollutants [11]. Previous studies used porous carbon, polymeric resin, clay, and agricultural waste as adsorbents, but the aforementioned adsorbents are inefficient and expensive materials [12,13,14,15,16]. Therefore, it is necessary to develop efficient adsorbents at a low cost. Zr-based MOFs showed excellent stability and high performance as adsorbents [17]. Qui et al. prepared UiO-66 (Zr-MOF) and achieved excellent adsorption of anionic dyes [18]. Zhan et al. synthesized UiO-66 and UiO-67 for the adsorption of acid orange 7 dye [19]. The MOFs are usually synthesized from toxic metal ions and organic linkers using toxic solvents [20]. So, it is challenging to prepare MOFs from less toxic inorganic metals and organic linkers using harmless solvents. An ecofriendly Zr-based biocompatible MOF (MIP-202) was synthesized using aspartic acid as a bio-organic linker. As both the inorganic Zr^4+^ metal and the organic aspartic acid linker of MIP 202 are harmless to the human body, so, water was used as a safe solvent for the synthesis of Zr-Bio-MOF for the complete production of an environmentally friendly adsorbent material. The synthesized biocompatible Zr-Bio-MOF was employed as an adsorbent of trimethoprim antibiotic from an aqueous polluted solution. Trimethoprim is one of the commonly used antibiotics due to its ability to treat microbial infections caused by a wide range of bacteria [21]. The frequent release of wastewater from pharmaceutical industries as well as domestic wastewater to water streams without an appropriate treatment results in the existence of trimethoprim in the aquatic environment [22]. The formation of bacteria resistant to antibiotics due to the spread of antibiotics in the aquatic environment has attracted wide attention [23]. Therefore, it is urgent to remove antibiotics from the wastewater before the discharge to water bodies.

In this study, biocompatible Zr-Bio-MOF was synthesized and analyzed using various physico-chemical techniques to evaluate its morphology, chemical composition, and chemical structure. The synthesized Zr-Bio-MOF was employed as an adsorbent of trimethoprim antibiotic from polluted wastewater. The effects of operating parameters such as pH, adsorbent dose, initial trimethoprim concentration, and reaction time on the removal efficiency of trimethoprim were studied to elucidate the kinetics of the adsorption process.

## 2. Materials and Methods

### 2.1. Materials

Zirconium tetrachloride (ZrCl_4_, 99.5%) and L-aspartic acid (C_4_H_7_NO_4_, LA, 99.0%) were obtained from Strem Chemicals Inc. (Newburyport, MA, USA) and Sigma Aldrich Co., Ltd. (St. Louis, MO, USA), respectively. Deionized water was used as the solvent for the preparation of all chemicals solutions. Ethanol (99.9%, CH_3_OH) was purchased from Fisher Scientific. All chemicals obtained were used directly without further purification.

### 2.2. Synthesis of Bio-MOF (MIP-202)

In a 25 mL screw-capped jar, 0.7 g (5.26 mmol) of L-Aspartic acid was dispersed in 5 mL of deionized water. The dispersed powder of L-Aspartic acid was then sonicated in an isothermal oven at 40 °C for 40 min to give a well-dispersed solution of the bio-organic ligand. In another 25 mL screw-capped jar, 0.57 g (2.465 mmol) of ZrCl_4_ was completely dissolved in 5 mL of deionized water under stirring for 5 min. Then, the solution of ZrCl_4_ was added dropwise to the L-Aspartic solution with a stirring rate of 200 rpm. Subsequently, the mixture was transferred to a 25 mL round-bottom flask and heated at 373 K using reflux and the mixture was stirred for 12 h. After cooling the mixture to room temperature, the precipitate was collected by centrifugation at 7000 rpm, washed vigorously with deionized water and ethanol for three days, and subsequently dried in air. The air-dried Bio-MOF sample was transferred to a vacuum chamber. The chamber was first evacuated at room temperature for 5 h. Finally, the sample was dried under vacuum at 70 °C for 12 h yielding the powder of MIP-202.

### 2.3. Experimental Procedures

The adsorption experiments were performed in a Pyrex beaker (250 mL) including 100 mL of trimethoprim solution. The solution was mixed using a magnetic stirrer (150 rpm) to achieve continuous contact between the MIP-202 adsorbent and the trimethoprim antibiotic molecules, and samples were withdrawn every 15 min. The influences of adsorbent dose, pH, and initial trimethoprim concentration were investigated by changing the adsorbent dose from 0.1 g/L to 1.5 g/L, solution pH was varied from 1 to 11, and the initial trimethoprim concentration from 2.5 mg/L to 20 mg/L. All experiments were performed at room temperature (25 ± 4 °C). The removal efficiency percentage of trimethoprim antibiotic (R (%)) and the adsorption capacity of the Bio-MOF (q_t_) were measured according to Equations (1) and (2).

(1) $$R\;(\%)=\frac{C_{o}-C_{t}}{C_{o}}\times 100$$

(2) $$q_{t}\;(\frac{\mathrm{mg}}{g})=\frac{C_{o}-C_{t}\;}{w}\times \;V$$

where R (%) is the removal efficiency of trimethoprim, C_o_ is the initial concentration of trimethoprim, C_t_ (mg/L) is the trimethoprim concentration at time (t), V(L) is the solution volume, and w (g) is the adsorbent weight.

### 2.4. Analytical Methods

The concentration of trimethoprim antibiotic was detected using high pressure liquid chromotography HPLC (Agilent 1200 series, Santa Clara, CA, USA), according to the literature [21].

The X-ray spectra was recorded using a Shimadzu XRD-6100 with Cu–Kα radiation (λ = 1.54 Å) to identify the crystalline structure. The morphology of the synthesized Bio-MOF was investigated using scanning electron microscopy (SEM, JEOL JSM-6010LV) equipped with energy dispersive X-ray (EDX) spectroscopy to specify the chemical composition of the synthesized Bio-MOF. The surface area and the pore size distribution of the prepared MOF were determined using a Belsorp-max automated apparatus. The prepared Bio-MOF was degassed at 200 °C for 5 h before measurement. To assess the chemical states of the prepared Bio-MOF, an X-ray photoelectron spectroscopy (XPS, Thermo Fisher Scientific, Waltham, MA, USA) analysis with X-ray Al kα radiation was used. Photoluminescence spectra were recorded using an Agilent Cary Eclipse Fluorescence Spectrophotometer. The thermal stability of the synthesized Bio-MOF was evaluated using thermogravimetric analysis (TGA, TGA-50, Shimadzu, Kyoto, Japonia). The temperature was increased from 25 °C to 1000 °C using nitrogen, and the weight of the sample was measured during the temperature rise. The heating rate and flow rate were 10 °C/min and 40 mL/min, respectively.

### 2.5. Langmuir and Freundlich Adsorption Isotherm Models

The relation between the amount of the adsorbed pollutant on the adsorbent’s surface at equilibrium and the effluent concentration were investigated using adsorption isotherms [24]. Langmuir and Freundlich isotherm models were employed to analyze the experimental data of the trimethoprim adsorption onto the Bio-MOF surface.

The Langmuir isotherm model is based on some assumptions, such as all adsorption sites are identical in size and shape, the number of vacant sites is equal, and the vacant site can be only occupied by one molecule [25]. Moreover, the Langmuir model assumes that the same amount of heat energy is released during adsorption. Langmuir’s model can be described in linear form as shown in Equation (3).

(3) $$\frac{C_{e}}{q_{e}}=\frac{C_{e}}{q_{m}}+\frac{1}{K_{L\;}q_{m}}$$

where q_e_ is the amount of trimethoprim adsorbed per 1.0 g of the adsorbent at equilibrium (mg/g), q_m_ is the maximum amount of trimethoprim adsorbed on the unit mass of the adsorbent (mg/g), K_L_ is the Langmuir adsorption constant (L/mg), and C_e_ is the concentration of trimethoprim at equilibrium (mg/L).

The Freundlich isotherm model can be applied to the heterogeneous adsorption process [26]. The Freundlich model can be represented in the linear form as depicted in Equation (4).

(4) $$\mathrm{Ln}(q_{e})=\ln(K_{f})+\frac{1}{n}\;\ln(C_{e})$$

where K_f_ and n are the Freundlich isotherm constants, K_f_ refers to the adsorption capacity of the adsorbent, and n describes the favorability of the adsorbent.

### 2.6. Adsorption Kinetics

The adsorption mechanism and dynamics were studied using kinetic models, such as the pseudo-first and second order models [27]. The kinetic behavior of the adsorption reaction can be understood using pseudo-first order model as shown in Equation (5):

Ln(q_e_ − q_t_) = ln(q_e_) − K_1_ × t (5)

where q_e_ and q_t_ are the amount of trimethoprim adsorbed on the unit mass of the adsorbent at equilibrium and time (t), respectively. K_1_ is the constant of the pseudo-first order model (min^−1^).

The pseudo-second order model can be presented as shown in Equation (6):

(6) $$\frac{t}{q_{t}}=\;\frac{1}{K_{2}}\;\times \;\frac{1}{q_{e\;}{}^{2}}+\;\frac{t}{q_{e}}$$

where k_2_ is the constant of the pseudo-second order model (g/mg/min).

## 3. Results and Discussion

### 3.1. Synthesis and Characterization of Bio-MOF

**Figure 1:**
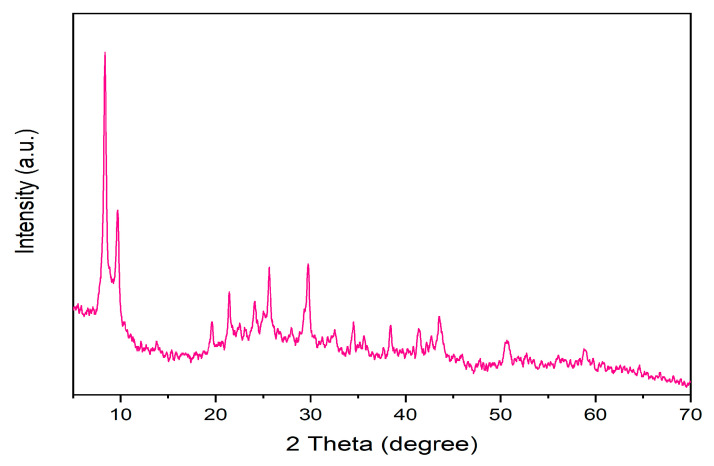
PXRD diffraction patterns of dry and soaked prepared Bio-MOF (MIP-202).

The synthesized MIP-202 crystalline structure is shown by X-ray diffraction (XRD) in Figure 1. The PXRD pattern of the synthesized MOF exhibited distinct peaks at 8.5°, 9.9°, 13.9°, 19.9°, and 21.7°, which were assigned to the (111), (200), (222), (420), and (440) planes of MIP-202. The other peaks were imputed to the diffraction planes of MIP-202, according to the literature. The stability of the synthesized MIP-202 was evaluated by soaking MIP-202 in water for 24 h. The diffraction peaks of the soaked particles were identical to the peaks of dry particles affirming the stability of MIP-202 in water. Therefore, the synthesized MOF can be employed in water treatment applications.

**Figure 2:**
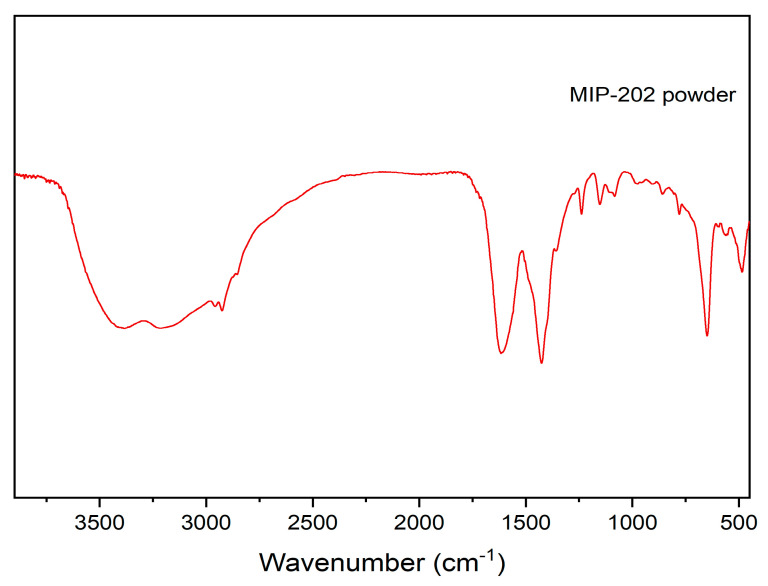
FTIR spectra of prepared Bio-MOF (MIP-202).

FTIR spectra, shown in Figure 2, for the synthesized MIP-202 demonstrates the characteristic peaks of the Bio-MOF. The peaks at 1652 cm^−1^ and 1568 cm^−1^ were attributed to the C-O and C-C stretching modes, respectively. The double characteristic peaks at 3490 cm^−1^ and 3380 cm^−1^ were appointed to the asymmetric and symmetric vibration of the −NH_2_ groups, whereas the peaks at 1590 cm^−1^ and 1340 cm^−1^ were allocated to the −NH_2_ bending vibration and the C−N stretching modes, respectively [3,5].

**Figure 3:**
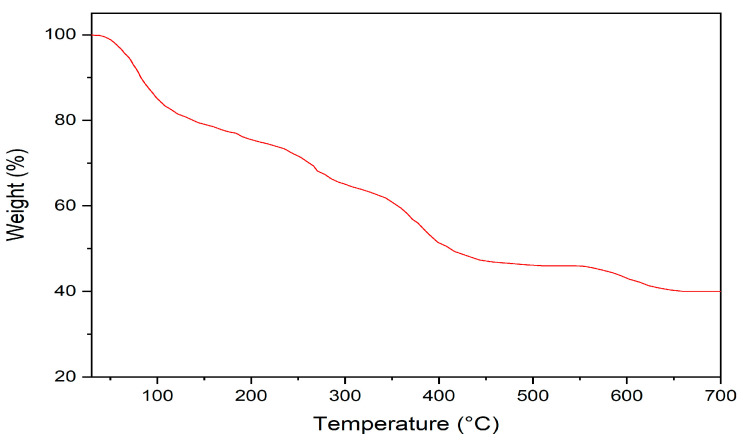
TGA profile of the prepared Bio-MOF (MIP-202).

Thermogravimetric analysis (TGA) was conducted in air to evaluate the thermal stability of the synthesized Bio-MOF, as shown in Figure 3. A weight loss ratio of about 20% observed at a temperature of nearly 240 °C was due to the loss of residual water molecules and atmospheric gases trapped into the pores. The increase in temperature from 240 °C to 450 °C increased the weight loss percentage to 56% as a result of the decomposition of organic matter.

**Figure 4:**
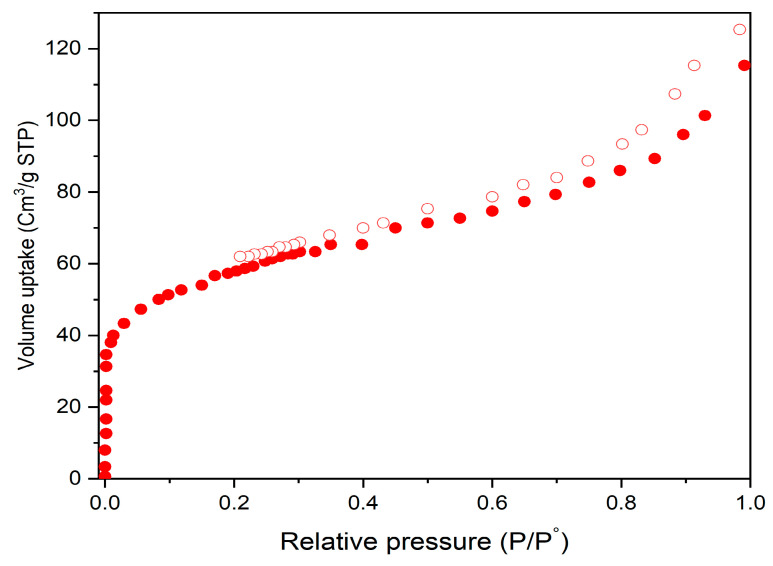
N_2_ adsorption−desorption isotherms for the BET surface area of the prepared Bio-MOF (MIP-202).

The surface area and pore size distribution are shown in Figure 4. The estimated surface area of the synthesized Bio-MOF was nearly 49.5 m^2^ g^−1^. The wide pore size distribution confirmed the high porosity of the prepared MIP-202.

**Figure 5:**
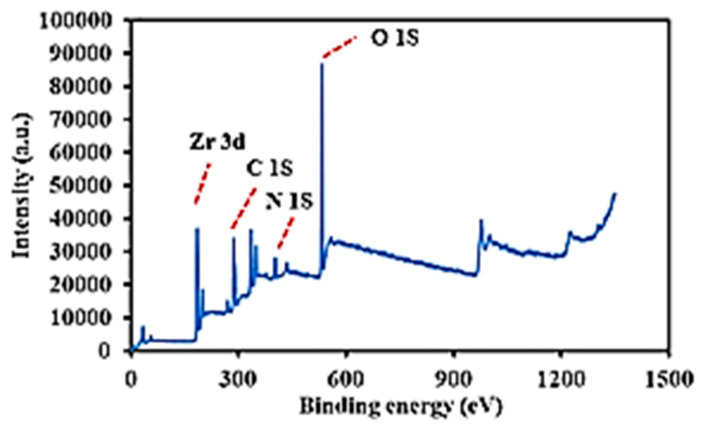
XPS analysis of the prepared Bio-MOF (MIP-202).

The chemical states of the synthesized MIP-202 were recorded by an X-ray photoelectron spectroscopy (XPS) as shown in Figure 5. The XPS survey spectra affirmed the existence of Zr, C, O, and N at the prepared Bio-MOF. The peak around 288.36 eV was ascribed to C 1S. The peak around 401.37 eV was due to the presence of N 1S. The peak at 529.65 eV was appointed to O 1S. The peak at 182.38 eV was due to Zr 3d3/2, whereas the peak at 184.76 was attributed to Zr 3d5/2.

**Figure 6:**
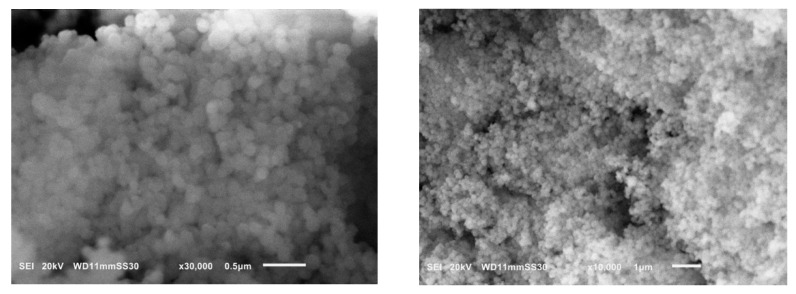
SEM images at different magnifications of the prepared Bio-MOF (MIP-202), synthesized at 110 ℃ for 18 h in pure water.

The morphology of the synthesized Bio-MOF is shown in Figure 6; it was evident from SEM imaging that the material had a sphere-like structure. The size of particles ranged from 70 to 80 nm, which confirmed the high surface area of the prepared MIP-202.

The EDX analysis confirmed the simultaneous existence of C, O, N, and Zr. The small chlorine content was due to the high interaction between chloride ions and ^+^NH_3_ through the pores of the prepared Bio-MOF during the preparation process.

### 3.2. Effect of Contact Time and Initial Trimethoprim Antibiotic Concentration

**Figure 7:**
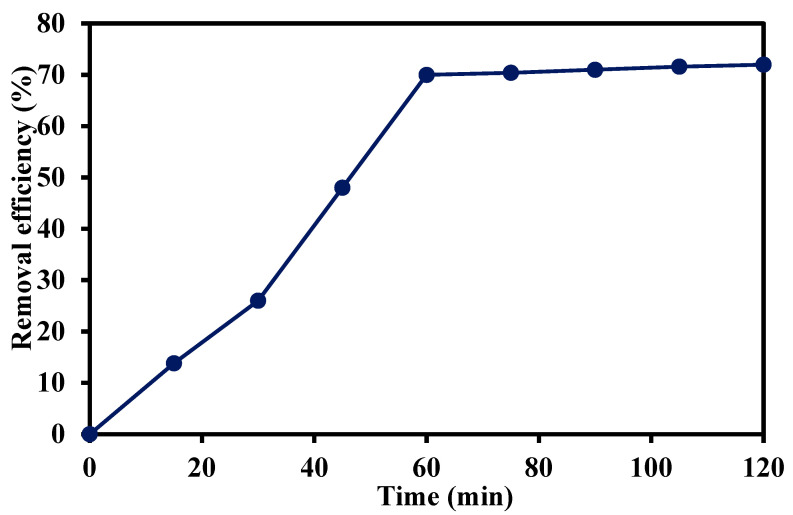
Effect of contact time on the removal efficiency of trimethoprim antibiotic at pH of 7, initial trimethoprim concentration of 10 mg/L, and adsorbent dose of 0.5 g/L.

The effect of contact time on the removal efficiency of trimethoprim antibiotic is illustrated in Figure 7. Contact time is one of the major factors that influences adsorption efficiency. An initial experiment was conducted at a contact time of 120 min, initial trimethoprim concentration of 5 mg/L, pH of 7, and adsorbent dose of 0.5 g/L. The removal rate of trimethoprim was high in the first 60 min due to the availability of vacant binding sites on the adsorbent’s surface [28]. However, the removal efficiency of trimethoprim antibiotic did not significantly increase by raising the contact time from 60 min to 120 min, as the binding sites on the adsorbent’s surface became filled with the trimethoprim molecules [29]. All subsequent experiments were conducted at a contact time of 60 min.

**Figure 8:**
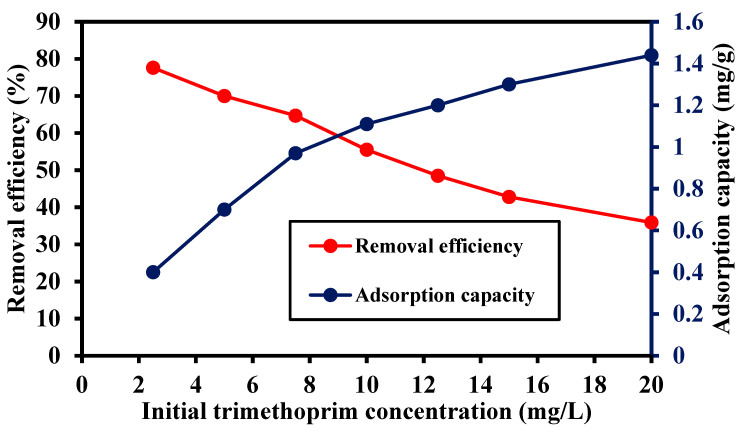
Effect of initial trimethoprim concentration on the removal efficiency of trimethoprim antibiotic and the adsorption capacity of Bio-MOF, pH of 7, contact time of 60 min, and adsorbent dose of 0.5 g/L.

The influence of the initial trimethoprim concentration on the removal percentage onto the synthetized MIP202 and its adsorption capacity is depicted in Figure 8. The removal efficiency of trimethoprim antibiotic decreased from 77.6% to 35.9% by increasing the initial trimethoprim concentration from 2.5 mg/L to 20 mg/L, at a pH of 7, a reaction time of 60 min, and adsorbent dose of 0.5 g/L. The decrease in trimethoprim removal efficiency was due to the unavailability of binding sites on the adsorbent’s surface to uptake trimethoprim molecules in the case of higher concentrations of trimethoprim. However, the adsorption capacity of trimethoprim increased from 0.4 mg/g to 1.44 mg/g by raising the initial trimethoprim concentration from 2.5 mg/L to 20 mg/L onto the synthetized MIP202. This behavior may be due to the increase in trimethoprim concentration, which acts as a driving force to overcome the mass transfer resistance of trimethoprim molecules between aqueous phases and solid resulting in the increase in equilibrium sorption [30]. 

### 3.3. Effect of MIP-202 Dosage

**Figure 9:**
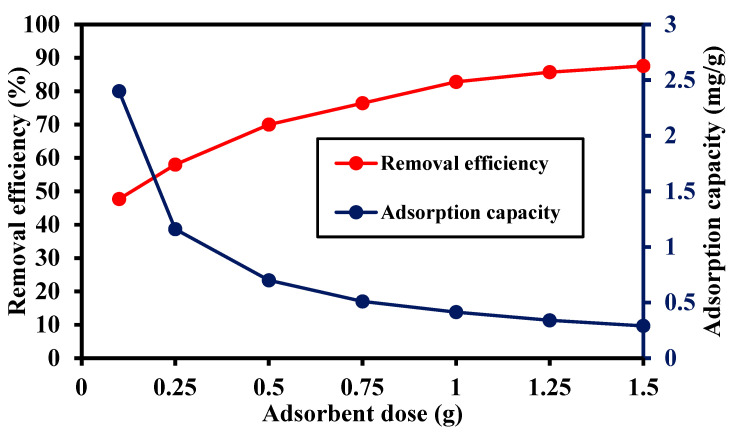
Effect of adsorbent dose on the removal efficiency of trimethoprim antibiotic and the adsorption capacity of Bio-MOF, pH of 7, contact time of 60 min, and adsorbent dose of 0.5 g/L.

The effect of the adsorbent dose on the removal efficiency of trimethoprim antibiotic and the adsorption capacity of the Bio-MOF is shown in Figure 9. The rise in adsorbent dose from 0.1 g/L to 1.5 g/L increased the removal efficiency of trimethoprim antibiotic from 47.7% to 87.6%. This may be imputed to the existence of available adsorption sites on the adsorbent’s Bio-MOF surface due its efficient high surface area [29]. However, the adsorption capacity of trimethoprim declined from 2.4 mg/g to 0.29 mg/g by raising the adsorbent dose from 0.1 g/L to 1.5 g/L. At a high dosage of adsorbents, an aggregation and/or overlapping of the adsorbent may take place resulting in the decline of the surface area available for antibiotic adsorption as well as the increase in diffusion path length [31].

### 3.4. Effect of Solution pH

**Figure 10:**
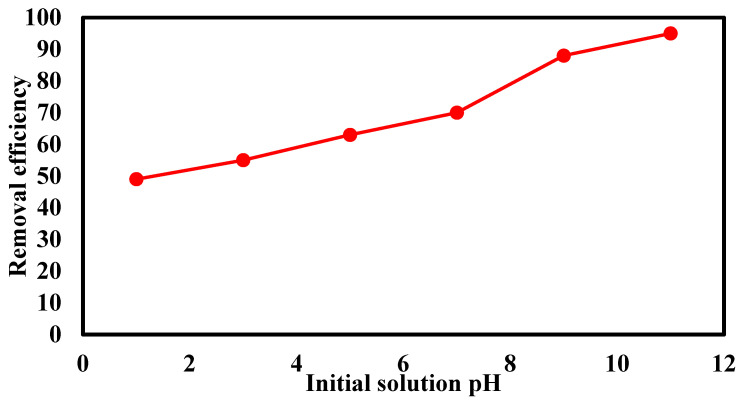
Effect of solution pH on the removal efficiency of trimethoprim antibiotic, contact time of 60 min, and adsorbent dose of 0.5 g/L.

Figure 10 shows the effect of pH on the removal efficiency of trimethoprim antibiotic at the initial trimethoprim concentration of 5.0 mg/L, adsorbent dose of 0.5 g/L, and contact time of 60 min. The solution pH greatly influenced the removal efficiency of trimethoprim, as the solution pH changed, the adsorbent’s surface was changed as well as the charge of the trimethoprim molecules [26]. The pka1 and pka2 of trimethoprim antibiotic were 3.2 and 7.2, respectively, so trimethoprim became negatively charged at a pH higher than 7.2 and positively charged at a pH lower than 3.2 [21]. The highest removal efficiency of trimethoprim recorded as 95 % was achieved at a pH of 11. The increase in removal rate at high pH values may be due to the attraction forces between the adsorbent and the trimethoprim molecules.

### 3.5. Langmuir and Freundlich Adsorption Isotherm Models

**Figure 11:**
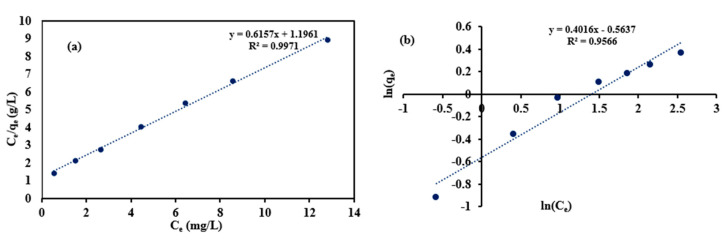
The linear adsorption isotherms of the trimethoprim antibiotic onto Bio-MOF: (**a**) Langmuir model and (**b**) Freundlich model.

In the Langmuir isotherm model, the relation between C_e_ and C_e_/q_e_ was fitted to estimate the Langmuir isotherm constants as shown in Figure 11a. The values of q_m_ and K_L_ were estimated to be 1.62 mg/g and 0.315 L/g, respectively. The value of the coefficient of determination (R^2^) was 0.99 affirming the appropriateness of the Langmuir isotherm model to describe the adsorption of trimethoprim antibiotic onto the prepared the Bio-MOF surface.

In the Freundlich isotherm, the relation between ln(qe) and ln(Ce) in Figure 11b was fitted and the slope and the intercept of the aforementioned relation refer to the values of 1/n and ln(K_f_), respectively. The value of 1/n (0.4) was lower than 1 confirming that trimethoprim antibiotic was favorable to be adsorbed on the prepared Bio-MOF surface. The R2 in the case of Freundlich model was 0.95. The R2 in the case of the Langmuir model was higher than the R^2^ using Freundlich model, so the Langmuir model better described the experimental data confirming the chemical interaction between trimethoprim antibiotic and the function groups at the MIP-202 Bio-MOF.

### 3.6. Pseudo-First and Second Order Kinetics Models

**Figure 12:**
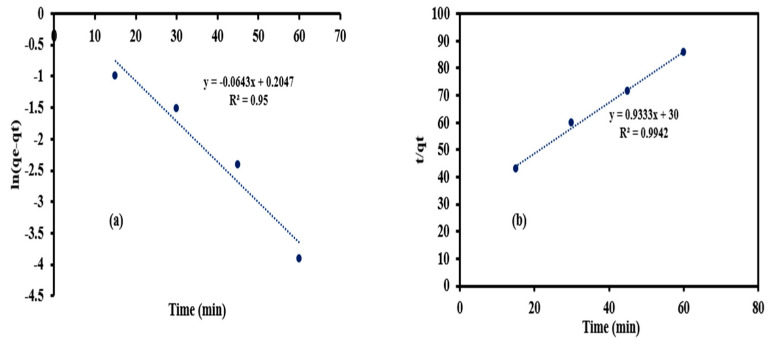
(**a**) Pseudo-first order and (**b**) Pseudo-second order kinetics of the adsorption of trimethoprim antibiotic on the Bio-MOF.

The pseudo-first order constant is the slope of the relation between log (q_e_ − q) and time as shown in Figure 12a. The values of K_1_ and q_e_ were 0.0643 min^−1^ and 1.23 mg/g, respectively. The high value of R^2^ (0.95) indicated that the pseudo-first order kinetic model was suitable to describe the adsorption process of trimethoprim antibiotic onto the prepared MIP-202 Bio-MOF. The values of q_e_ and K_2_ can be determined by measuring the slope and intercept of the relation between (t/q_t_) and time in the case of pseudo-second order kinetic model as demonstrated in Figure 12b. The values of K_2_ and q_e_ were 0.029 g/mg/min and 1.07 mg/g. The experimental equilibrium adsorption capacity (0.72) was near to the estimated equilibrium adsorption capacity from the pseudo-second order model confirming the applicability of this model. Moreover, the high value of R^2^ (0.99) affirmed the suitability of the pseudo-second order model to describe the adsorption process. The value of R^2^ in the case of the pseudo-first order model was lower than the R2 of pseudo-second order model, and the difference between the experimental and theoretical adsorption capacity at equilibrium was lower in the case of the pseudo-second order kinetic model confirming the appropriateness of second order kinetic model to describe the adsorption process of trimethoprim antibiotic onto the prepared MIP-202 Bio-MOF, confirming the physico-chemical adsorption process.

## 4. Conclusions

An eco-friendly Zr-based MOF was synthesized using aspartic acid as an organic linker and water as a solvent. The chemical composition and structure as well as the morphology and thermal stability were evaluated using various analyses. The synthesized Bio-MOF was employed as an adsorbent for the removal of trimethoprim from aqueous solutions. The effects of various operating parameters such as pH, contact time, initial trimethoprim concentration, and adsorbent dose on the removal efficiency of trimethoprim were investigated. The increase in contact time above 60 min did not significantly enhance the removal efficiency. The increase in initial trimethoprim concentration decreased the removal efficiency of trimethoprim, whereas the increase in adsorbent dose improved the removal efficiency. The highest removal efficiency of trimethoprim antibiotic was achieved at a pH of 11. The Langmuir model better described the adsorption process than the Freundlich model. The pseudo-second order model was suitable to describe the adsorption process confirming the chemical interactions between the studied water pollutant and the prepared MIP-202 Bio-MOF.

## Data Availability

Not applicable.
